# Gut microbiota-mediated pain sensitization: mechanisms and therapeutic implications

**DOI:** 10.3389/fpain.2025.1626515

**Published:** 2025-07-03

**Authors:** Minghe Zhao, Ling Zhang, Zhihui Liu

**Affiliations:** ^1^ Department of Anesthesiology, Baotou Central Hospital, Baotou, China; ^2^ Baotou Clinical Medical College, Inner Mongolia Medical University, Baotou, China; ^3^ Department of Gastroenterology, Baotou Central Hospital, Baotou, China

**Keywords:** pain, gut microbiota, microbiome-gut-brain axis, sensitization, neuroinflammation, GPR43, TLR2

## Abstract

Emerging evidence has illuminated the pivotal role of gut microbiota in modulating pain sensitivity through bidirectional gut-brain interactions. Current research demonstrates that gut microbial communities significantly influence pain perception by regulating both central and peripheral sensitization mechanisms across various pain modalities. This review synthesizes current knowledge on the mechanisms underlying gut microbiota-mediated pain sensitization, encompassing: (1) cross-talk within the microbiome-gut-brain axis, (2) regulatory effects of microbial metabolites on central and peripheral sensitization pathways, and (3) bioactive compounds derived from gut microbiota that participate in pain modulation. Furthermore, we systematically evaluate the therapeutic potential of microbiota-targeted interventions including probiotic supplementation, fecal microbiota transplantation, and dietary modifications in pain management. To advance this promising field, future investigations should prioritize three key directions: establishing causal relationships through rigorous verification, accelerating clinical translation of preclinical findings, and developing personalized microbial-based therapeutic strategies.

## 1 Introduction

Recent years have witnessed growing interest in the regulatory role of gut microbiota in pain modulation at the intersection of neuroscience and microbiology. Gut microbial communities establish complex bidirectional interactions with their host through three principal pathways: metabolite production, immune modulation, and neural signaling transmission. Clinical investigations have demonstrated strong associations between gut dysbiosis and various chronic pain disorders, particularly neuropathic pain, fibromyalgia, and irritable bowel syndrome (1–3). The advancement of high-throughput sequencing and metabolomics technologies has empowered researchers to progressively unravel the molecular mechanisms through which gut microbiota modulates central and peripheral sensitization via the gut-brain axis (4–6). Notably, microbiota-derived metabolites such as short-chain fatty acids (SCFAs), bile acids, and tryptophan catabolites can directly or indirectly engage receptors (e.g., GPR43, TLR4, TRP channels) on neuronal and immune cells, inducing neuroinflammation and synaptic plasticity alterations that ultimately drive pain sensitization (7–9). Furthermore, gut dysbiosis may compromise blood-brain barrier (BBB) integrity, which facilitates the translocation of pro-inflammatory factors (including IL-1β and TNF-α) into the central nervous system, thereby exacerbating glial cell activation and central sensitization (10–12). While these findings provide novel insights into pain mechanisms, three critical knowledge gaps persist:1. the causal relationships between specific dysbiotic taxa and pain phenotypes remain unestablished, 2. the spatiotemporal specificity of key metabolic pathways requires systematic elucidation, and 3. the clinical translatability of microbiota-based interventions needs comprehensive evaluation. This review synthesizes recent advances in gut microbiota-mediated pain sensitization by delineating three mechanistic dimensions: gut-brain axis signaling, metabolite-mediated regulation, and central/peripheral sensitization mechanisms, while critically assessing the therapeutic potential of probiotics, fecal microbiota transplantation (FMT), and dietary interventions. By integrating preclinical and clinical evidence, we aim to establish a theoretical framework for developing microbiota-targeted strategies in pain management.

### 1.1 Disclaimer and future perspectives

It is critical to acknowledge that the therapeutic recommendations proposed in this review are constrained by current limitations in the field. **First**, the causal relationships between specific microbial taxa and pain phenotypes remain inferential due to insufficient longitudinal human data and interventional studies. **Second**, the heterogeneity in host genetics, diet, and comorbidities across clinical cohorts complicates the generalizability of microbiota-based interventions. **Third**, the dynamic spatiotemporal regulation of microbial metabolic pathways and their interactions with host receptors (e.g., GPR43, TLR2/4) requires further mechanistic dissection.

### 1.2 Future research should prioritize

Causal validation: Utilizing germ-free animal models coupled with targeted bacterial colonization to establish microbial causality in pain sensitization. Clinical translation: Conducting large-scale randomized controlled trials (RCTs) to evaluate microbiome-targeted therapies (e.g., probiotics, FMT) in stratified pain subtypes. Personalized approaches: Integrating multi-omics data (metagenomics, metabolomics, host epigenomics) to develop precision microbial therapeutics. Long-term safety: Assessing longitudinal effects of microbiota modulation on neurological and immunological functions. Mechanistic depth: Elucidating strain-specific effects and receptor crosstalk (e.g., GPR43-TLR4 synergy) using single-cell transcriptomics and spatial metabolomics.

## 2 The gut microbiota and the gut-brain axis

The gut microbiota refers to microbial communities colonizing the host's gastrointestinal tract, with human intestinal flora being predominantly composed of bacteria. These microbial populations, historically estimated to outnumber human somatic cells by a 10:1 ratio, play indispensable roles in maintaining host homeostasis (1). The gut microbiota exhibits dynamic fluctuations throughout the host's lifespan, modulated by both endogenous and exogenous factors including genetic predisposition (13), sex differences (14), geographical environment (15), dietary habits and nutritional patterns (16), as well as individual physiological status (17). These microbial communities interface with human physiology through multiple pathways, particularly via the gut microbiota-brain axis—a bidirectional communication network. This conceptual framework, which describes the reciprocal signaling between the gastrointestinal tract and the central nervous system (CNS) (18), is mechanistically underpinned by neuroregulation, neurotransmitter activity, and neuroimmune interactions. This axis encompasses five major physiological systems: immune, endocrine, enteric nervous, autonomic nervous, and central nervous systems. Through integrated neuroanatomical, neurochemical, and molecular signaling mechanisms, it coordinates bidirectional communication between the CNS and gut microbiota communities (4).

## 3 Mechanisms underlying gut microbiota-mediated central sensitization

Central sensitization refers to a maladaptive plasticity within the CNS characterized by amplified neuronal responsiveness to nociceptive signaling, resulting in heightened pain perception to both noxious stimuli and normally innocuous inputs; Mechanistically, neuroinflammatory cascades—whether triggered by primary neural inflammation or secondary to tissue injury—constitute the core driver of pain-related central sensitization. This pathophysiological process manifests through sustained glial activation, dysregulated cytokine networks, and maladaptive synaptic potentiation within nociceptive pathways (19). The gut microbiota drives neuroinflammatory processes through metabolite production (notably SCFAs) that activate immune cells such as macrophages and T lymphocytes. Mechanistically, SCFAs engage G protein-coupled receptors (e.g., GPR43) on these immune cells, triggering the release of pro-inflammatory cytokines including interleukin-1β (IL-1β), tumor necrosis factor-α (TNF-α), and IL-17. These signaling molecules subsequently translocate across the BBB, inducing microglial activation within the CNS that ultimately precipitates central sensitization (20, 21).

### 3.1 Microbiota-derived metabolite-mediated GPR43 signaling pathway

**Figure 1:**
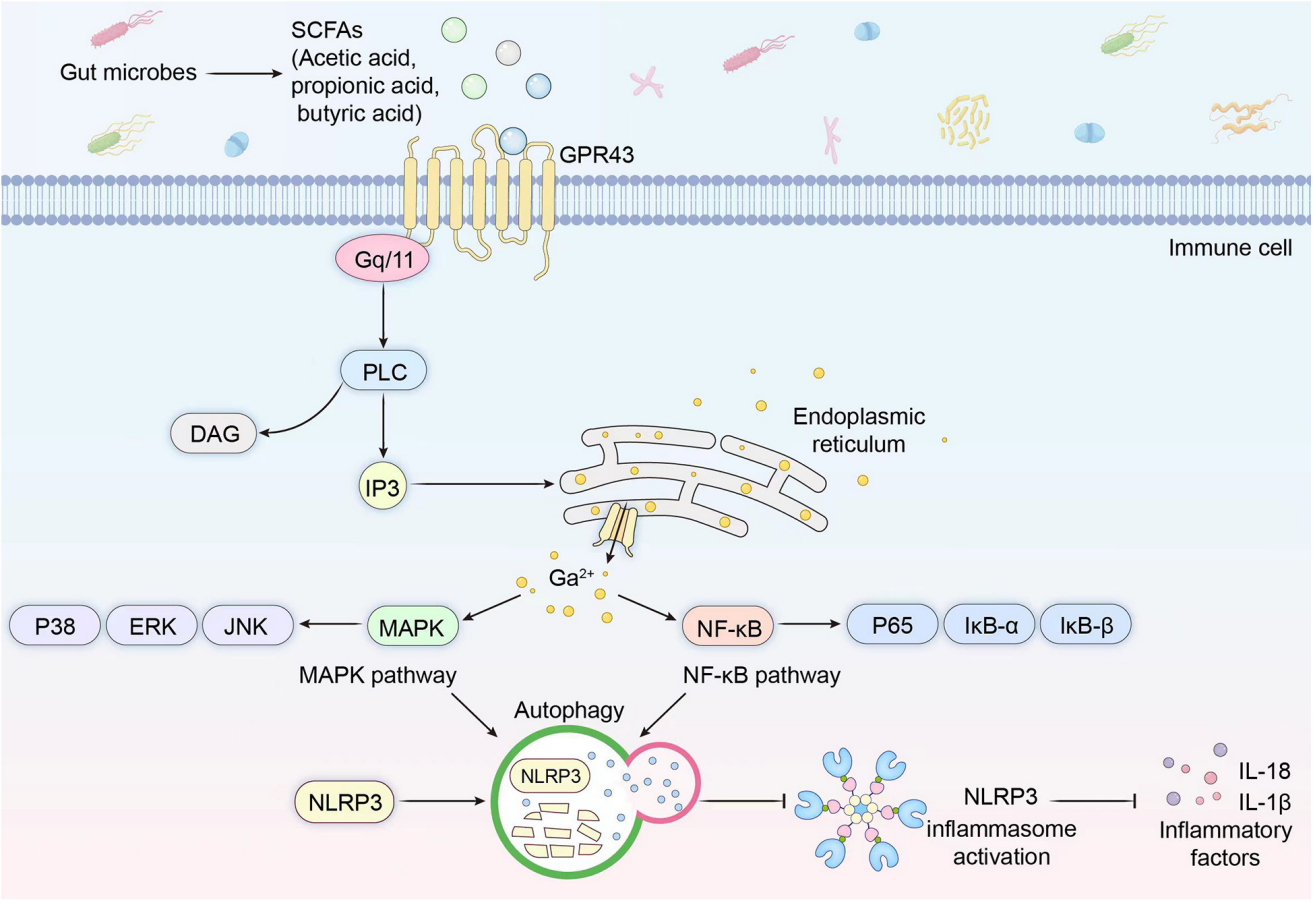
GPR43 pathway. The schematic illustrates the molecular mechanism by which gut microbiota-derived short-chain fatty acids (SCFAs), including acetate, propionate, and butyrate, modulate neuroinflammatory signaling through the GPR43 pathway.

While SCFAs are generally recognized for their anti-inflammatory properties, they paradoxically exhibit context-dependent pro-inflammatory effects through GPR43-mediated immune cell activation under specific pathological contexts. A representative example is acetate's capacity to engage GPR43 on neutrophils, potentiating their chemotaxis, oxidative burst, and cytokine production (22). The metabolic pathway of SCFAs involves their entry into systemic circulation, where they engage G protein-coupled receptors (GPR43) expressed on immune cell surfaces. Notably, GPR43 represents one of the primary receptors for SCFAs, which serves as a critical signaling cascade initiating immune cell activation and subsequent systemic immunomodulatory effects (23). Following SCFAs-GPR43 binding, receptor activation triggers the recruitment of Gq/11 protein subunits. These activated subunits subsequently stimulate phospholipase C (PLC), catalyzing the generation of inositol trisphosphate (IP3) and diacylglycerol (DAG). IP3 binds to endoplasmic reticulum-localized IP3 receptors, inducing Ca²^+^ release that elevates intracellular calcium levels. This calcium surge sequentially activates both the nuclear factor-kappa B (NF-κB) and MAPK signaling pathways, which facilitate PKA-mediated ubiquitination of the NLRP3 inflammasome. The ubiquitinated NLRP3 complex undergoes autophagic degradation, thereby suppressing inflammasome activation and ultimately inhibiting the change of pro-inflammatory cytokines (7, 24, 25) (Figure 1).

### 3.2 Microbiota-derived metabolite-mediated TLR2 signaling axis

**Figure 2:**
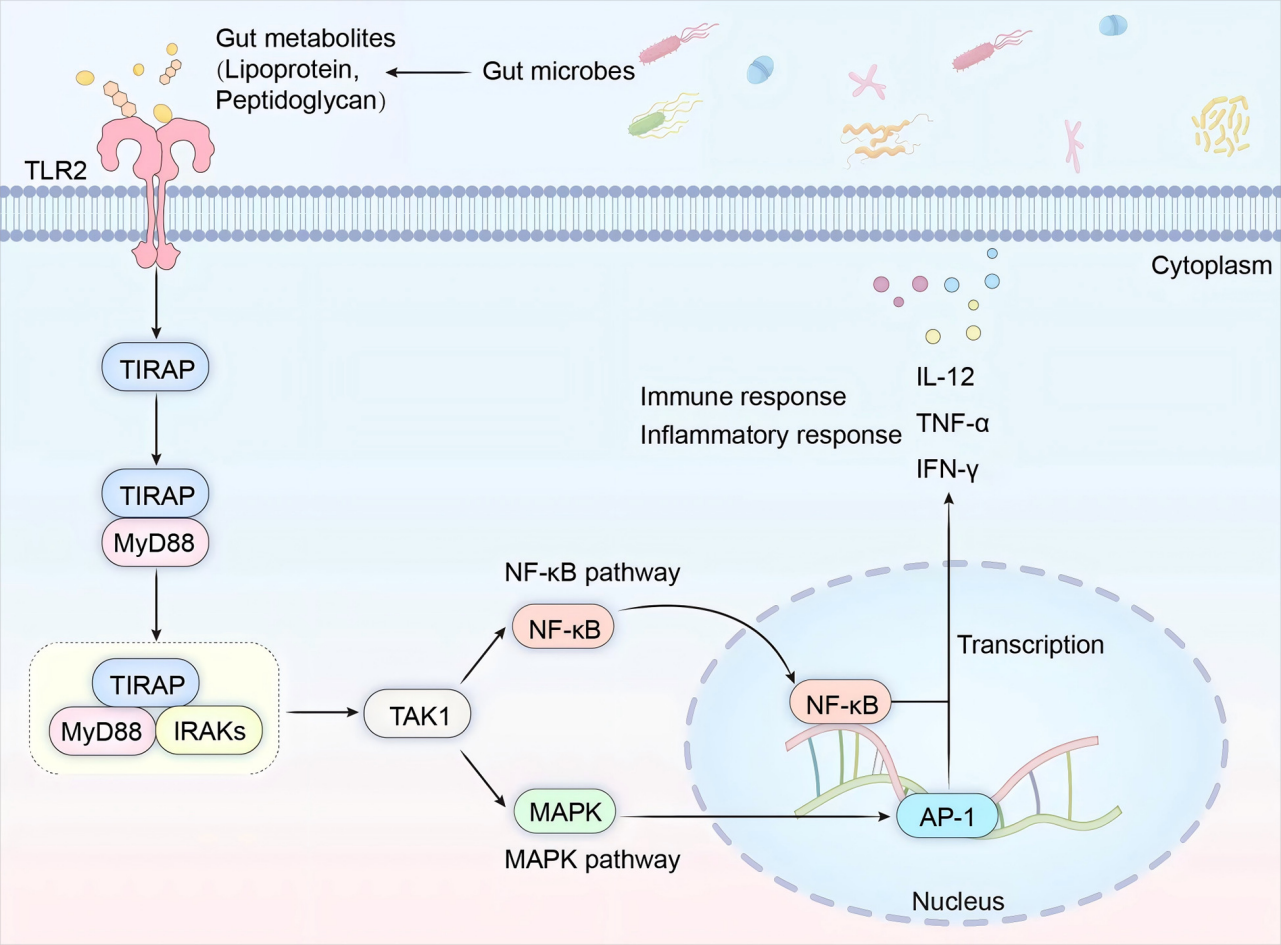
TLR2 pathway. This schematic delineates the molecular cascade triggered by enteric pathogens through Toll-like receptor 2 (TLR2)-mediated signaling, culminating in immune activation and inflammation.

Beyond the well-characterized GPR43 pathway, gut microbiota-derived metabolites can alternatively activate immune cells through Toll-like receptor 2 (TLR2) engagement, while also triggering the release of pro-inflammatory cytokines. This dual-receptor mechanism is exemplified by specific microbial metabolites (e.g., lipoteichoic acid) that bind TLR2 on macrophages, subsequently initiating myeloid differentiation primary response 88 (MyD88)-dependent signaling cascades and nuclear factor-κB (NF-κB) activation (26). The gut microbiota metabolizes indigestible dietary fibers through fermentation, generating diverse bioactive compounds including SCFAs, lipoproteins, and peptidoglycans. Notably, specific metabolites derived from Gram-positive bacteria—particularly lipoproteins and peptidoglycans—serve as ligands for TLR2 on intestinal immune cells, initiating TLR2-mediated signaling via MyD88/PI3K pathways. Mechanistically, TLR2 forms functional heterodimers with either TLR1 or TLR6 to recognize pathogen-associated molecular patterns (PAMPs) such as lipopeptides. Upon ligand binding, TLR2 dimerization triggers sequential recruitment of Toll/IL-1 receptor domain-containing adaptor protein (TIRAP) and MyD88. The MyD88 complex subsequently activates IL-1 receptor-associated kinases (IRAKs), with IRAK4 and IRAK1 undergoing phosphorylation. Activated IRAKs bind tumor necrosis factor receptor-associated factor 6 (TRAF6), forming a signaling complex that activates NF-κB and mitogen-activated protein kinase (MAPK) pathways through transforming growth factor β-activated kinase 1 (TAK1). NF-κB activation involves dissociation from its inhibitor IκB, followed by nuclear translocation to initiate transcription of pro-inflammatory genes. This process upregulates key inflammatory mediators (TNF-α, IL-6, IL-1β) that orchestrate immune cell activation, particularly in B lymphocytes, macrophages, and dendritic cells (27–29). These activated immune cells subsequently sustain the release of pro-inflammatory cytokines, notably TNF-α, IL-12, and IFN-γ, thereby amplifying inflammatory cascades through autocrine and paracrine signaling loops (30) (Figure 2).

### 3.3 Microbiota-Derived metabolite-mediated GPR109A (HCAR2) signaling pathway

Emerging evidence highlights the role of G-protein coupled receptor 109A (GPR109A/HCAR2) as a critical mediator in gut-brain-pain interactions. This receptor, primarily activated by the endogenous ketone body β-hydroxybutyrate (BHB) and microbial-derived butyrate, exerts anti-nociceptive effects through modulation of neuroinflammation. Upon activation, GPR109A suppresses NF-κB signaling and inhibits NLRP3 inflammasome assembly in microglia and astrocytes, thereby reducing pro-inflammatory cytokine release (e.g., IL-1β, TNF-α) in the central nervous system (CNS) (31). Notably, BHB—produced during ketogenesis or by gut microbial metabolism—acts as a high-affinity endogenous ligand for GPR109A. Preclinical studies demonstrate that GPR109A agonism (e.g., by monomethyl fumarate) significantly alleviates mechanical allodynia and thermal hyperalgesia in neuropathic pain models by attenuating spinal glial activation and neuronal hyperexcitability (32, 33). Furthermore, gut dysbiosis-induced reductions in butyrate production impair GPR109A signaling, exacerbating neuroinflammatory cascades and central sensitization (34). Given its dual regulation by host-derived ketone bodies and microbiota-derived SCFAs, GPR109A represents a promising therapeutic target for pain disorders linked to gut-brain axis dysregulation.

### 3.4 Pro-inflammatory factors infiltrate the central nervous system

The BBB, composed of brain capillary endothelial cells, pericytes, and astrocytes, serves to safeguard the CNS against blood-borne toxins and pathogens (35). Under neuroinflammatory conditions, pro-inflammatory cytokines compromise BBB integrity through multiple mechanisms, ultimately enhancing permeability. For instance, TNF-α and IL-1β activate receptors on endothelial cells to induce redistribution of tight junction proteins, thereby increasing barrier permeability (10, 36). Elevated BBB permeability facilitates pro-inflammatory cytokine translocation into the CNS (10). Upon penetration into the CNS, pro-inflammatory mediators activate glial cells, initiating pathological cascades that modulate nociceptive processing.

### 3.5 Glial cell activation and sensitization represent pivotal cellular mechanisms in neuroinflammatory cascades

CNS-infiltrating pro-inflammatory cytokines bind to glial cell receptors, triggering glial activation. These activated glial cells acquire the capacity to release glutamate and other neurotransmitters, modulating synaptic activity. The released glutamate contributes to postsynaptic currents, thereby amplifying excitatory signaling. Experimental evidence demonstrates that optogenetic modulation of cerebellar Bergmann glial cells—employing archaerhodopsin-T or channelrhodopsin-2—enables bidirectional regulation of glutamate release, directly establishing glial control over synaptic transmission (37). Glial cells exert pivotal regulatory roles in maintaining synaptic homeostasis through excitatory synapse modulation. For instance, elevated ammonium levels suppress neuronal activity by compromising glial function, while inhibition of glial glutamine synthetase attenuates the downregulation of excitatory transmission (38). Following entry into the CNS via compromised BBB regions or active transport, TNF-α triggers microglial activation in the spinal dorsal horn. The microglia-derived TNF-α binds to neuronal TNFR1 receptors, activating the p38 MAPK signaling pathway, which induces phosphorylation of Nav1.8 sodium channels, thereby augmenting neuronal excitability. Concurrently, TNF-α suppresses Gamma-aminobutyric acid (GABA)ergic interneuron function, reducing inhibitory synaptic transmission, ultimately culminating in central sensitization and chronic pain (39). IL-1β enters the CNS via the meningeal lymphatic system and binds to astrocytic IL-1R1 receptors in the spinal dorsal horn, inducing ATP release. The released ATP activates neuronal P2X4 receptors, triggering presynaptic glutamate release and enhancing postsynaptic responses through phosphorylation of the NMDA receptor NR2B subunit. This mechanism induces long-term potentiation (LTP) of C-fiber-evoked potentials in the spinal dorsal horn, which is critically implicated in neuropathic pain (40). Interleukin-6 (IL-6) accesses the cerebrospinal fluid (CSF) via the choroid plexus, activating the IL-6R/gp130 complex on astrocytes to initiate the JAK2-STAT3 signaling pathway. Following nuclear translocation, STAT3 upregulates glial synthesis of COX-2 and PGE2. The released PGE2 binds to neuronal EP2 receptors, downregulating potassium-chloride cotransporter 2 (KCC2) expression, which induces intracellular Cl^−^ accumulation and loss of GABAergic inhibition. This mechanism critically underlies pain hypersensitivity in chemotherapy-induced peripheral neuropathy models (41, 42). IFN-γ enters the CNS through active transport, activating IFN-γ receptors (IFN-γR) on spinal microglia to induce indoleamine 2,3-dioxygenase (IDO) expression. IDO metabolizes tryptophan into kynurenine, which suppresses neuronal Kv4.2 potassium channels via the aryl hydrocarbon receptor (AhR), thereby enhancing action potential firing frequency. This mechanism not only exacerbates pain hypersensitivity but also induces pain-associated anxiety and depressive behaviors through IDO activation in the anterior cingulate cortex (ACC) (43).

**Figure 3:**
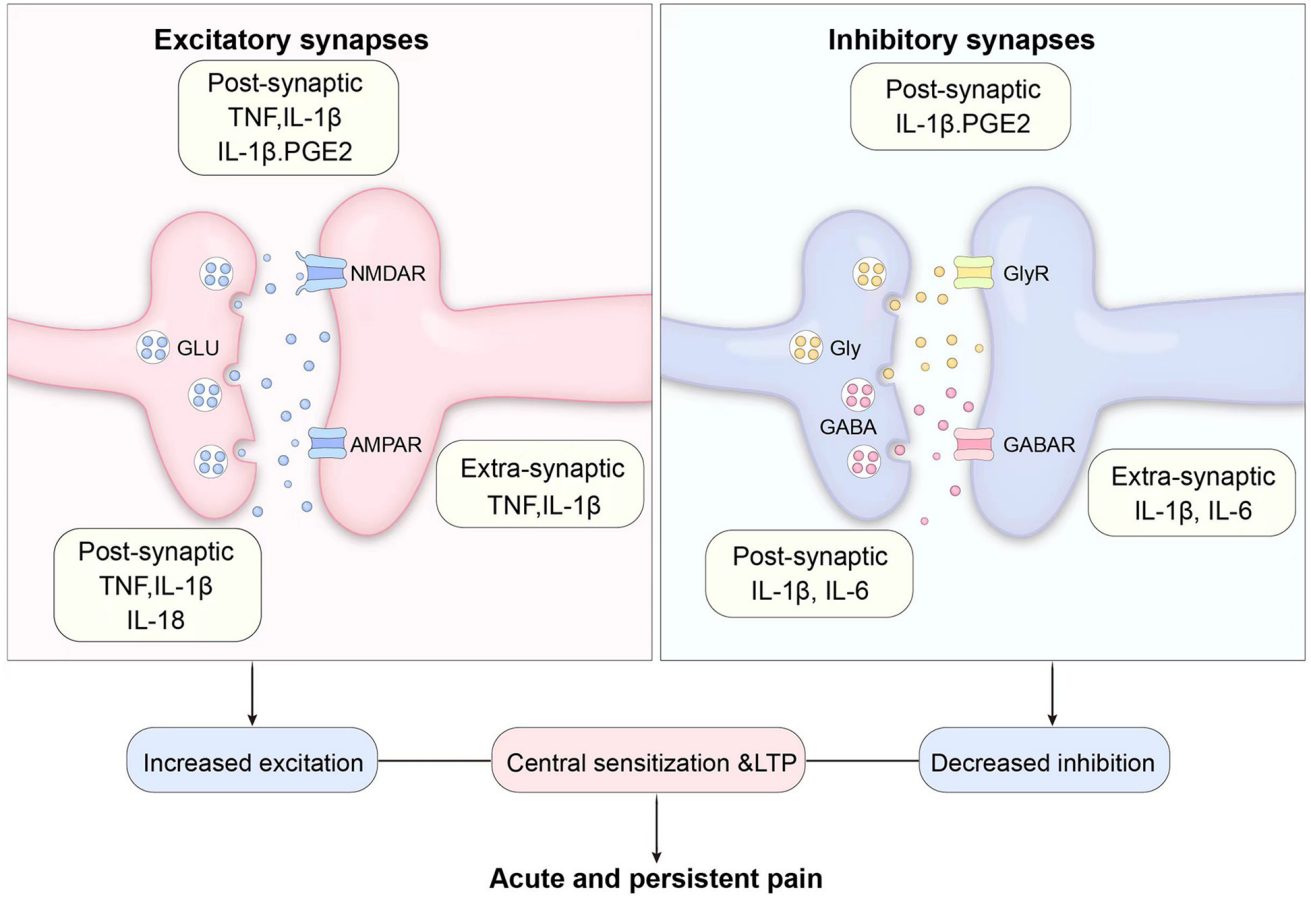
Co-facilitation of pain sensitization. This schematic illustrates the dual modulation of excitatory and inhibitory synapses by inflammatory mediators, leading to drive pain hypersensitivity.

Activated microglia release increased inflammatory mediators, thereby amplifying neuroinflammation (11, 44). The C-C motif chemokine ligand 2(CCL2) enters the spinal dorsal horn through compromised BBB, binds to CCR2 receptors on microglia, and triggers NLRP3 inflammasome activation with subsequent IL-18 release. IL-18 acts on IL-18 receptors (IL-18R) at primary afferent nerve terminals, enhancing membrane expression and function of transient receptor potential vanilloid 1 (TRPV1) channels to promote calcium influx and substance P release. Concurrently, CCL2 directly activates dorsal root ganglion (DRG) neurons, augmenting the voltage-gated sodium channel 1.7 (Nav1.7) sodium currents via the PKC*ε* pathway, thereby increasing spontaneous discharges (45). Activated glial cells additionally generate pro-inflammatory cytokines such as TNF-α, IL-1β, and chemokines including CXC motif chemokine ligand 1 (CXCL1), synergistically amplifying neuroinflammatory exacerbation. In parallel, glia-derived mediators induce imbalanced synaptic transmission—enhanced glutamatergic signaling and/or diminished GABA-ergic synaptic efficacy—which collectively promote central sensitization development, ultimately driving pain hypersensitivity (46) (Figure 3).

Moreover, cerebral endothelial cells, pericytes, microglia, astrocytes, and infiltrating immune cells receive peripheral signals originating from the gastrointestinal (GI) tract. Activation of these cell populations drives neuroinflammatory progression, while the gut microbiota critically regulates microglial immune function and maturation (2).

### 3.6 Crosstalk between receptor signaling pathways in pain sensitization

Gut flora metabolites mediate nociceptive sensitization through the activation of multiple receptors (e.g., GPR43, TLR2/4, ASIC3), and these pathways do not operate independently, but rather form an interacting network through the following mechanisms:

#### 3.6.1 Receptor synergy and signaling integration

TLR4-P2X3 complex formation:

Secondary bile acids activate the ERK1/2 pathway via TLR4, upregulate P2X3 receptor expression in DRG neurons and promote complex assembly, enhancing ATP-induced calcium inward flow (↑Ca²^+^ inward flow) and synergistically amplifying neuronal hyperexcitability (55).

TLR4-TRPV1/TRPA1 coactivation:

LPS synergistically induces chronic pain by simultaneously upregulating the expression of TRPV1 (PKC*ε*-mediated phosphorylation) and TRPA1 (direct binding to intracellular structural domains) via the TLR4/NF-κB pathway and lowering the activation threshold of both (67).

GPR43-TLR4 immunoregulation synergizes:

SCFAs inhibit HDAC activity and upregulate Kv1.2 potassium channel expression (↓ neuronal excitability) via GPR43; whereas LPS promotes pro-inflammatory factor release (↑ neuroinflammation) via TLR4, and the two form a bi-directional regulatory network via epigenetic and inflammatory pathways (56, 95).

#### 3.6.2 Shared downstream signaling nodes

NF-κB pivotal role:

TLR4 activation activates NF-κB through the MyD88/IRAK/TRAF6 cascade (26). GPR43 regulates the NLRP3 inflammasome through the PLC-IP3-Ca²^+^-NF-κB axis (7). NF-κB integrates multi-pathway signaling and collectively upregulates the expression of nociception-related ion channels such as Nav1.7, TRPV4, and ASIC1/3 (53, 65, 80).

MAPK pathway crossover:

TLR4 activation of p38 MAPK promotes Nav1.8 phosphorylation (39). GPR43 regulates inflammatory factor release through the Ca²^+^-MAPK axis (25). Multiple pathways converge on MAPK to amplify spinal dorsal horn neuronal sensitization (46).

#### 3.6.3 Metabolite-mediated receptor interactions

Bidirectional regulation of SCFAs:

Propionic acid inhibits HDAC (antinociception) via the TLR4 nonclassical binding site but activates pronociception via TRPV1/TRPV2 (56, 62).

Homeostatic effects of tryptophan metabolites:

Indole derivatives upregulate ASIC1a (pro-nociceptive sensitization) via AhR (79), but KYNA (tryptophan metabolite) inhibits neuronal excitability (anti-nociceptive sensitization) via GPR35 (98).

#### 3.6.4 Functional synergy at the cellular level

Neuro-immune interaction:

TLR4 activation induces CCL2 release→recruits macrophages to infiltrate DRG→enhances neuronal excitability via TNF-α/IL-6 (52). GPR43-mediated SCFAs inhibit microglia activation → reduce IL-1β/TNF-α release → indirectly inhibit central sensitization (20).

Synergistic regulation of synaptic plasticity:

Glial cell-released TNF-α inhibits GABAergic transmission (↓inhibitory synapses) while the TLR4 pathway enhances glutamatergic signaling (↑ excitatory synapses), which together promote central sensitization (39, 46).

In summary: Nociceptive sensitization is the result of dynamic interactions of multiple receptor pathways. the TLR family (TLR2/4/9) predominantly drives pro-nociceptive signaling, whereas GPR43/GPR35 and others play a protective role through anti-inflammatory and ion channel modulation. The spatiotemporal-specific cross-talk of these pathways (e.g., NF-κB/MAPK node integration, receptor heterodimerization) provides a theoretical basis for the development of multi-targeted analgesic strategies (Table 1).

**Table 1:** Standardized indexing of key receptors in public databases (data source: KEGG/uniProt version 2025).

Target	Function	KEGG ID	UniProt ID
GPR43	Signs of inflammation	map04064	O15552
TLR2	immune activation	map04620	O60603
ASIC3	pain sensitization	map04978	P78348

## 4 Mechanisms underlying gut microbiota modulation of peripheral sensitization

Peripheral sensitization refers to heightened hypersensitivity of nociceptive neurons and sensory receptors at peripheral nerve terminals, particularly within inflammatory or injured sites, thereby amplifying nociceptive responses to external stimuli.

### 4.1 Direct regulation of primary sensory neuron excitability in the DRG

At the peripheral level, gut microbiota modulates neuronal excitability in the peripheral nervous system (PNS). Specifically, microbiota-derived metabolites activate or sensitize pain-associated receptors and ion channels—including Toll-like receptors (TLRs), transient receptor potential (TRP) channels, GABA receptors, and acid-sensing ion channels (ASICs)—thereby directly regulating the excitability of primary sensory neurons within the DRG (47).

#### 4.1.1 Toll-like receptor 4 (TLR4)

As discussed earlier, the TLR2 pathway of microbiota-derived metabolites serves as a pivotal upstream pathway for central sensitization. Emerging evidence indicates that bacteria activate TLR-expressing sensory neurons; these neurons then utilize TLR4 to detect bacterial components, thereby eliciting nociceptive and inflammatory responses (48). Studies further demonstrate that Irinotecan-induced gastrointestinal dysfunction and pain are mediated through TLR4-dependent mechanisms (49). Emerging studies indicate that the probiotic DSF ameliorates chemotherapy-induced neuropathic pain through TLR4 expression modulation (50). These findings collectively demonstrate the critical involvement of TLR4 in nociception. Furthermore, immunohistochemical validation reveals TLR4 localization on the plasma membrane of DRG neurons. Mechanistically, TLR4 may modulate DRG neuronal function through transcriptional regulation of specific molecular factors, ultimately influencing axonal regeneration (51). Experimental evidence demonstrates that TLR4 activation in DRG neurons upregulates CCL2 expression, thereby inducing macrophage infiltration into DRG and modulating neuronal excitability (52). Furthermore, TLR4 enhances neuronal excitability by modulating the expression of Nav1.7. NF-κB, a pivotal downstream effector of the TLR4 signaling pathway, regulates neuronal excitability through transcriptional control of Nav1.7 expression (53). Beyond sodium channels, TLR4 may modulate Schwann cell function through activation of downstream signaling pathways including NF-κB and MAPK, promotes Schwann cell proliferation and migration, thereby impacting nerve regeneration processes (54). Secondary bile acids derived from gut microbiota metabolism, such as lithocholic acid, directly activate TLR4 on DRG neurons, increasing membrane expression of P2X3 receptors via the ERK1/2 signaling pathway. TLR4 forms a complex with P2X3 receptors, potentiating ATP-induced currents (Ca²^+^ influx), thereby resulting in neuronal hyperexcitability and visceral hypersensitivity. Co-administration of the TLR4 inhibitor CLI-095 and the P2X3 antagonist AF-353 synergistically alleviates bile acid-induced nociception (55). SCFAs, exemplified by propionate, bind to the extracellular domain of TLR4 on DRG neurons at non-canonical LPS-binding sites, thereby suppressing histone deacetylase (HDAC) activity. This HDAC inhibition elevates histone acetylation levels at the promoter region of the Kv1.2 potassium channel gene, augmenting its transcriptional expression, which enhances K^+^ efflux and reduces neuronal excitability (56). Indole and its derivatives (e.g., indole-3-acetic acid) produced by gut microbiota activate TLR4 on DRG neurons, triggering NADPH oxidase 2 (NOX2)-dependent reactive oxygen species (ROS) generation. These ROS oxidatively modify the Cys892 residue of the Nav1.8 sodium channel, delaying its inactivation, prolonging action potential duration, and increasing spontaneous firing frequency (57). Polyamines (e.g., putrescine) derived from gut microbiota metabolism directly bind to the intracellular TIR domain of TLR4, activating mTORC1 signaling to promote protein synthesis in DRG neurons. mTORC1 upregulates the expression of the acid-sensing ion channel 3 (ASIC3) and transient receptor potential ankyrin 1 (TRPA1) channels, thereby enhancing neuronal excitability induced by acid and oxidative stress. Pharmacological inhibition of TLR4 or mTOR with rapamycin significantly alleviates polyamine-induced mechanical hypersensitivity (58). Gut microbiota-derived CpG oligodeoxynucleotides (CpG-ODNs) activate Toll-like receptor 9 (TLR9) in DRG neurons, inducing heterodimerization of TLR4 and TLR9. The TLR4/9 heterodimer activates p38 MAPK via the IRAK1/TRAF6 signaling axis, enhancing voltage-gated calcium channel Cav3.2 currents, thereby facilitating neurotransmitter release and nociceptive transmission. Combined administration of TLR4-neutralizing antibodies and the TLR9 antagonist ODN2088 synergistically suppresses CpG-ODN-induced nociceptive effects (59). Collectively, these findings demonstrate that TLR signaling directly modulates the excitability of primary sensory neurons within the DRG, thereby driving peripheral sensitization.

#### 4.1.2 TRP channels

TRP channels constitute a class of non-selective cation channels that are evolutionarily conserved across diverse organisms, including *Homo sapiens*, fungi, and green algae such as *Chlamydomonas reinhardtii* (60). Emerging evidence indicates that multi-strain probiotics modulate TRP channel activity through regulation of gut microbiota metabolites, thereby alleviating migraine symptoms (61). This finding suggests an intrinsic association between TRP channels and nociception. Mechanistic investigations reveal that SCFAs, gut microbiota-derived metabolites, activate TRPV1 and TRPV2 channels. TRPV1 activation triggers Ca²^+^ and Na^+^ influx, inducing neuronal depolarization and high-frequency action potential firing, which amplifies nociceptive signal transmission and elevates neuronal excitability (62, 63). Gut microbiota metabolize tryptophan into indole and its derivatives (e.g., indole acrylic acid), which directly bind to the N-terminal cysteine residues (Cys621 and Cys665) of the TRPA1 channel, inducing conformational changes. TRPA1 activation triggers Ca²^+^ influx, enhances excitability of DRG neurons, and promotes neurogenic inflammation via release of calcitonin gene-related peptide (CGRP) and substance P (64). Secondary bile acids (e.g., deoxycholic acid) activate TLR4 on DRG neurons, upregulating transient receptor potential vanilloid 4 (TRPV4) channel expression via the NF-κB signaling pathway. TRPV4 channels are directly sensitized by bile acids, exhibiting increased open probability under hypotonic or mechanical stimulation, which drives Ca²^+^ influx and neuronal hyperexcitability. In cholestatic liver disease models, the TRPV4 antagonist HC-067047 significantly alleviates visceral pain (65). Polyamines (e.g., spermine, spermidine) derived from gut microbiota metabolism directly bind to the voltage-sensing domain (S4 segment) of the transient receptor potential melastatin 8 (TRPM8) channel, inhibiting cold- and menthol-evoked currents. By reducing TRPM8 open probability, polyamines diminish Ca²^+^ influx and suppress abnormal discharges in cold-sensing DRG neurons, as observed in cold allodynia associated with inflammatory bowel disease (66). Lipopolysaccharide (LPS) derived from Gram-negative bacteria activates the NF-κB pathway via TLR4 in DRG neurons, upregulating the expression of TRPA1 and TRPV1. LPS directly binds to the intracellular C-terminal domain of TRPA1, enhancing its sensitivity to endogenous ligands (e.g., 4-hydroxynonenal, 4-HNE), while concurrently phosphorylating the Ser800 residue of TRPV1 via protein kinase C epsilon (PKC*ε*), thereby lowering its activation threshold. This LPS-induced co-activation of TRPA1/TRPV1 culminates in neuronal hyperexcitability and chronic pain (67). Gut microbiota convert dietary ellagitannins into urolithin A (UroA), which directly binds to the pore region (Gly652 and Tyr658 residues) of the transient receptor potential canonical 5 (TRPC5) channel, antagonizing its non-selective cation currents. TRPC5 inhibition stabilizes the membrane potential of DRG neurons, thereby reducing spontaneous discharges and nociceptive transmission (68). Collectively, these findings demonstrate that TRP channels directly modulate the excitability of primary sensory neurons in the DRG, thereby driving peripheral sensitization.

#### 4.1.3 GABA

GABA serves as the primary inhibitory neurotransmitter in the CNS. By binding to its receptors, GABA evokes inhibitory postsynaptic potentials (IPSPs) that suppress neuronal excitability. As reviewed above, glial cells can modulate GABAergic tone, thereby contributing to central sensitization. Additionally, GABA may mediate peripheral sensitization through modulation of DRG neuronal activity (69). Tryptophan metabolites such as indole directly activate chloride channels by binding to the extracellular domain (non-canonical binding site) of the GABA-A receptor α5 subunit. The indole-evoked chloride current induces hyperpolarization of the resting membrane potential in DRG neurons, thereby reducing the frequency of action potential firing (70, 71). Secondary bile acids (e.g., lithocholic acid) activate TLR4 on the membrane of DRG neurons, inducing endocytosis of the γ2 subunit of GABA-A receptors and reducing surface receptor density. Concurrently, bile acids directly bind to the α1 subunit of GABA-A receptors, inhibiting GABA-mediated chloride currents and resulting in neuronal depolarization. Genetic ablation of TLR4 or pharmacological antagonism of the GABA-A α1 subunit with bicuculline reverses the pronociceptive effects of bile acids (72). Polyamines (e.g., putrescine) produced by gut microbiota traverse the blood-nerve barrier (BNB) to activate GABA-B receptors on DRG neurons, inhibiting the mTORC1 signaling pathway through Gβγ subunits. mTORC1 suppression reduces synaptic protein synthesis, thereby attenuating LTP and nociceptive sensitization (73). D-lactate (not L-lactate), a metabolite produced by gut microbiota, specifically binds to the *δ* subunit of GABA-Areceptors, enhancing channel open probability under low pH conditions (e.g., inflammatory states). The D-lactate-evoked chloride current suppresses the excitability of nociceptive DRG neurons expressing TRPV1, independent of the canonical GABA-binding site (74). Gut microbiota-derived metabolites, including SCFAs and bile acids, regulate the excitability of primary sensory neurons in the DRG by activating GABA receptors. These metabolites modulate GABA receptor expression and activity, thereby altering GABAergic inhibition and modifying neuronal excitability. Additionally, they influence GABA receptor functionality through phosphorylation state adjustments (75, 76).

#### 4.1.4 ASICs

ASICs, a class of extracellular proton (H^+^)-activated non-selective cation channels, are widely distributed throughout the central and peripheral nervous systems (77). Gut microbiota-derived SCFAs, such as butyrate and propionate, directly activate ASIC3 channels (exhibiting the highest pH sensitivity) by reducing extracellular pH. In DRG neurons, ASIC3 activation induces increased Na^+^ influx, triggering action potentials and enhancing neuronal excitability. Experimental studies demonstrate that genetic deletion of ASIC3 or pharmacological blockade with the antagonist APETx2 abolishes the pronociceptive effects of SCFAs (78). Furthermore, gut microbiota convert tryptophan into indole derivatives (e.g., indole-3-acetic acid), activating the AhR in DRG neurons. AhR signaling upregulates the transcriptional expression of ASIC1a, increasing channel surface density and amplifying acid-evoked currents. In diabetic neuropathy models, pharmacological inhibition of AhR attenuates ASIC1a-mediated hyperalgesia (79). LPS released by Gram-negative bacteria activates TLR4 in DRG neurons, triggering the NF-κB signaling pathway. NF-κB binds to the promoter regions of ASIC1/2/3 genes, upregulating their expression and enhancing acid-induced neuronal excitability. Elevated ASIC1/3 expression has been observed in DRG neurons from patients with irritable bowel syndrome (IBS) (80). Gut dysbiosis elevates histamine release, which activates H1 receptors in DRG neurons and potentiates ASIC3 activity via the PLC/PIP2 signaling pathway. ASIC3 co-localizes with H1 receptors on the neuronal membrane of DRG neurons. Histamine induces spontaneous firing by lowering the pH activation threshold of ASIC3 from 6.7 to 6.9 (81). Polyamines (e.g., putrescine, cadaverine) derived from gut microbiota metabolism directly bind to the extracellular domain of acid-sensing ion channel 1a (ASIC1a) near its proton-binding site. Through electrostatic interactions, polyamines stabilize the open conformation of ASIC1a, enabling channel activation under minimal pH reductions (pH 7.0→7.2). Cryo-electron microscopy (cryo-EM) structural analysis reveals hydrogen bonding between putrescine and the Glu220/Asp346 residues of ASIC1a (82). In addition to microbiota-derived metabolites, inflammatory and neuroinflammatory processes can modulate ASICs to mediate pain modulation (83).

### 4.2 Indirect modulation of primary sensory neuron excitability

On the other hand, gut microbiota metabolites indirectly modulate the excitability of primary sensory neurons in the DRG by activating non-neuronal cells (e.g., immune cells) to trigger the release of pro-inflammatory cytokines (e.g., IL-6, IL-1β, TNF-α), chemokines (e.g., CCL2), or anti-inflammatory cytokines (e.g., IL-4, IL-10) (84). Gut microbiota metabolites, including LPS and SCFAs, regulate the activation of microglia and astrocytes (85, 86), Reduced expression of tight junction proteins compromises BBB integrity, facilitating the entry of pro-inflammatory cytokines such as IL-1β and TNF-α into the CNS. This initiates inflammatory signaling cascades, ultimately shaping the brain's neuroinflammatory state and indirectly modulating food reward-related neurobehavioral signaling. Altered neurobehavioral signals may further regulate the excitability of primary sensory neurons in the DRG (87). Furthermore, activated microglia release IL-6 and TNF-α, which upregulate the expression of Nav1.7 sodium channels in DRG neurons via the TLR4/NF-κB signaling pathway, ultimately driving neuronal hyperexcitability (88). Gut microbiota-derived phenylacetylglutamine (PAGln) induces DNA damage and mitochondrial dysfunction, driving host cellular senescence via the adrenergic receptor-AMPK signaling pathway. The senescence process entails the release of inflammatory factors such as IL-6, which may indirectly modulate the excitability of primary sensory neurons in the DRG by activating non-neuronal cells (e.g., immune cells) (89). Gut microbiota-derived indole derivatives, such as indole-3-propionic acid, activate the AhR. AhR activation promotes the release of IL-6 and TNF-α, which subsequently activate the JAK-STAT3 signaling pathway in DRG neurons. This upregulates the expression of voltage-gated calcium channel Cav3.2, thereby enhancing neuronal excitability (90). Conversely, it promotes the release of the anti-inflammatory cytokine interleukin-10 (IL-10), which suppresses hyperexcitability in DRG neurons (91). Gut microbiota-derived histamine activates intestinal mast cells, triggering the release of IL-6 and TNF-α. These cytokines activate spinal microglia via the vagal-spinal pathway. Activated microglia release CCL2, recruiting peripheral monocytes to infiltrate the DRG. Through CCR2 receptor signaling, monocytes activate the ERK1/2 pathway within neurons, upregulating the expression of hyperpolarization-activated cyclic nucleotide-gated (HCN) channels, thereby enhancing spontaneous neuronal firing (92). All aforementioned mechanisms indirectly modulate the excitability of primary sensory neurons in the DRG via the neuro-immune-microbiome axis, culminating in the development of peripheral sensitization.

### 4.3 PAMPs

Furthermore, PAMPs derived from the gut microbiota are recognized as critical contributors to peripheral sensitization under chronic pain conditions. These PAMPs encompass cell wall components such as peptidoglycan (PGN), β-glucans, and LPS. Following localized release into circulation, these molecules interact with pattern recognition receptors on immune cells and sensory neurons within the DRG, thereby driving peripheral sensitization (26). On the one hand, PAMPs act on immune cells to release pro-inflammatory cytokines and chemokines, which indirectly activate or sensitize primary sensory neurons within the DRG. On the other hand, primary sensory neurons in the DRG can be directly activated or sensitized by PAMPs (5). Studies demonstrate that β-glucans produced by *Candida albicans* bind to dendritic cell-associated C-type lectin-1 (Dectin-1) in primary sensory neurons of the DRG. This Dectin-1-mediated phospholipase C-transient receptor potential vanilloid 1 (PLC-TRPV1) axis contributes to the nociceptive effects of β-glucans, thereby evoking pain responses in mice (93). Therefore, PAMPs derived from the gut microbiota induce neuronal hyperexcitability either by directly acting on primary nociceptive neurons or indirectly via immune cell activation, thereby driving peripheral sensitization.

## 5 The role of gut microbiota-derived metabolites in pain modulation

### 5.1 SCFAs

The gut microbiota ferments indigestible dietary fibers into SCFAs, primarily propionate, butyrate, and acetate. In addition to their involvement in central sensitization-associated pathways as previously described, SCFAs modulate diverse leukocyte functions by activating free fatty acid receptors 2 and 3 (FFAR2/3), regulating the production of cytokines (IL-2, IL-6, IL-10, and TNF-α) and chemokines (e.g., CCL2) (94). TNF-α plays a critical role in peripheral sensitization during neuropathic pain. Butyrate, functioning as an HDAC inhibitor, reduces TNF-α production via HDAC suppression, thereby attenuating pain responses in peripheral nerve injury models. Therefore, SCFAs modulate pain through both receptor-mediated mechanisms and histone acetylation-dependent epigenetic regulation (95).

### 5.2 Bile acids (BA)

The gut microbiota serves as a critical determinant in regulating BA metabolism (96), BAs induce analgesia by activating the G protein-coupled bile acid receptor (TGR5) in peripheral macrophages, mechanistically through the release of endogenous opioids (8). Accumulating evidence indicates that the gut microbiota plays a pivotal role in the biosynthesis of kynurenic acid (KYNA) (97), G protein-coupled receptor 35 (GPR35) is upregulated in DRG neurons. Activation of GPR35 KYNA reduces neuronal excitability *in vitro* and induces dose-dependent analgesia *in vivo* (98).

### 5.3 KYNA

KYNA, a metabolite of the kynurenine pathway, modulates neurotransmission and pain perception. KYNA is produced via tryptophan metabolism, primarily synthesized in astrocytes through the enzymatic actions of kynurenine 3-monooxygenase (KMO) and kynurenine aminotransferase (KAT). KYNA regulates neurotransmission by antagonizing α7 nicotinic acetylcholine receptors (α7nAChRs) and *N*-methyl-D-aspartate receptors (NMDARs) (99), KYNA exerts antihyperalgesic effects in the CNS through modulation of glutamatergic neurotransmission (9). KYNA alleviates pain by antagonizing NMDARs, thereby reducing excessive glutamatergic activation. Dysregulated KYNA levels are closely associated with chronic pain states such as fibromyalgia, while disturbances in its metabolic pathways may exacerbate neuroinflammation and amplify pain perception.

### 5.4 GABA

The inhibitory neurotransmitter GABA can be synthesized by *Lactobacillus*, *Bifidobacterium dentium*, and *Bifidobacterium* species through enzymatic decarboxylation of glutamate via glutamate decarboxylase-B (GAD-B). Daily oral administration of *Bifidobacterium* species modulates sensory neuronal activity in a rat model of visceral hypersensitivity with fecal retention (100). Recent studies have demonstrated that GABA receptors are expressed in DRG neurons. GABA receptor activation induces depolarization of most sensory neuronal somata. However, due to the filtering effect at T-junctions of nociceptive fibers, this generates a net inhibitory effect on nociceptive transmission, ultimately alleviating neuropathic and inflammatory pain (101).

### 5.5 Neuromodulators

Studies have demonstrated that multiple neuromodulators participate in the neuroplasticity of pain perception during the transition from acute to chronic pain, thereby inducing plasticity-related alterations at both molecular and network levels (102). Endogenous pain dysregulation, a hallmark of chronic pain, appears to emerge as one of the consequences of neuroplastic remodeling within the CNS (103). Within the CNS, descending pain modulatory pathways process nociceptive signals through the neurotransmitters norepinephrine (NE) and serotonin (5-HT). NE suppresses pain perception, whereas 5-HT exhibits bidirectional modulation—both inhibiting and facilitating nociceptive processing (104). The gut microbiota modulates the availability of circulating tryptophan and influences 5-HT synthesis in the brain (105) (Tables 2, 3).

**Table 2:** Key Gut Microbiota-derived metabolites and their roles in pain sensitization.

Metabolite	Mechanism of action	Pain modulation	References
Short-Chain Fatty Acids (SCFAs)	Activate GPR43/FFAR2 on immune cells, induce NF-κB/MAPK signaling; inhibit HDACs to regulate epigenetic modifications.	Pro-/anti-inflammatory effects (context-dependent), modulate neuronal excitability via TRP channels.	(7, 24, 25, 62, 95)
Bile Acids (BAs)	Activate TGR5 to release endogenous opioids; bind TLR4 on DRG neurons, enhancing P2X3/TRPV4 expression.	Analgesia (via TGR5) or hyperalgesia (via TLR4).	(8, 55, 96)
Kynurenic Acid (KYNA)	Antagonize NMDA/α7nAChRs; activate GPR35 to suppress neuronal excitability	Central antihyperalgesia, reduces neuroinflammation.	(9, 98, 99)
GABA	Bind GABA-A/B receptors on DRG neurons; modulate chloride currents to inhibit action potentials.	Suppresses peripheral nociception and neuropathic pain.	(70, 72, 100)
Indole Derivatives	Activate AhR/TRPA1; induce ROS generation via TLR4-NOX2 axis.	Promotes hyperalgesia (ROS) or analgesia (AhR-dependent).	(57, 64, 79)

**Table 3:** Molecular cascade linking Gut Microbiota-derived metabolites to pain sensitizatio.

Metabolite	Receptor/target	Signaling pathway	Key molecular events	Cellular effect	Pain modulation	References
SCFAs (e.g., hydrocyanic acid)	GPR43 (immune cells) TLR4 (DRG neurons) TRPV1/TRPV2 (DRG neurons)	Gq/11-PLC-IP₃/DAG NF-κB Ca²^+^ instream flow	↑ Ca²^+^ release → activation of NF-κB/MAPK → NLRP3 ubiquitination/degradation HDAC inhibition → ↑ histone acetylation → ↑ Kv1.2 expression Channel activation → Ca²^+^/Na^+^ endocytosis	↓ Inflammasome activation → inhibits IL-1β/TNF-α release ↑ K^+^ efflux → ↓ neuronal excitability Neuronal depolarization → ↑ action potential frequency	Anti-inflammatory/analgesic (inhibits central sensitization) Analgesic (inhibits peripheral sensitization) Pro-nociceptive (induces peripheral sensitization)	(7, 24, 25) (56) (62, 63)
Bas (e.g., litho bile acids)	TLR4 (DRG neurons) TGR5 (macrophage)	ERK1/2 Endogenous opioid release	↑ P2X3 membrane expression → TLR4/P2X3 complex formation Activation of TGR5 → β-endorphin release	↑ ATP-induced current (Ca²^+^ inward flow) → neuronal hyperexcitability Inhibition of injurious signaling	Pro-analgesia (visceral pain sensitization) Analgesic	(55) (8)
KYNA	NMDA receptor/α7nAChR (CNS) GPR35 (DRG neurons)	Glutamatergic signaling antagonism G protein coupling	Blockade of NMDA receptors → ↓Ca²^+^ inward flow Activation of GPR35 → ↓ voltage-gated calcium channel activity	↓ Synaptic plasticity (e.g., LTP) → ↓ central sensitization ↓Neurotransmitter release → ↓ neuronal excitability	Antinociceptive (central analgesia) Analgesic	(9, 99) (98)
Indole derivatives (e.g., IAA)	TLR4 (DRG neurons) AhR (DRG neurons)	NOX2-ROS Transcriptional regulation	↑ ROS generation → Nav1.8 oxidative modification (Cys892) AhR activation → ↑ASIC1a expression	Delayed Nav1.8 inactivation → ↑ spontaneous discharge ↑ Acid-induced current → ↑ neuronal excitability	Pain promotion (Mechanical pain sensitization) Promoting Pain (Diabetic Neuropathy)	(57) (79)
GABA	GABA-A/receptor (DRG neurons)	Cl-channel activation	Cl- inward flow → neuronal hyperpolarization	↓ Action potential frequency	Analgesia (inhibits peripheral injury transmission)	(70, 72, 100)

## 6 Therapeutic potential of microbiota-targeted interventions in pain management

### 6.1 Probiotic interventions in postoperative pain management

Probiotics alleviate postoperative pain by modulating gut microbiota composition and attenuating neuroinflammation. Surgical procedures and anesthesia disrupt intestinal microbial homeostasis, resulting in heightened inflammatory responses. Probiotics restore microbial equilibrium by replenishing beneficial bacteria such as *Bifidobacterium* and *Lactobacillus* species. Through suppression of pro-inflammatory mediators (e.g., IL-6, TNF-α), probiotics mitigate postoperative neuroinflammatory processes, thereby reducing nociceptive sensitization (106, 107); Furthermore, probiotics can be integrated into multimodal analgesia strategies to reduce dependence on opioid medications (108). Butyrate, SCFAs, modulates gut microbiota composition and attenuates inflammatory responses, thereby alleviating postoperative pain. By regulating the functionality of the enteric nervous system, butyrate suppresses nociceptive signaling. This mechanistic insight provides a novel rationale for employing probiotic interventions in postoperative pain management (6). In two clinical trials of 120 abdominal surgery and 80 postoperative orthopedic patients, the experimenters intervened with a probiotic complex (containing Bifidobacterium and Lactobacillus) and L. rhamnosus GG for 4 weeks, respectively, from 7 days preoperatively to 14 days postoperatively, and the results of the trial indicated that the probiotic intervention resulted in a reduction in postoperative pain scores by 30%–50%, as well as a significant reduction in pro-inflammatory factors (IL-6,TNF-α) levels (↓38%–41%) and reduced opioid dependence (↓35%) (106, 108). These clinical trials reinforce the above points.

### 6.2 Clinical trials of FMT for chronic pain management

FMT significantly reduced patients’ pain scores on the Numerical Rating Scale (NRS), improved the Widespread Pain Index (WPI), and alleviated Symptom Severity (SS) scores. Post-FMT treatment, patients exhibited significant increases in serum levels of 5-HT and GABA, alongside a marked reduction in glutamate concentrations. The therapeutic effects of FMT demonstrated durability, persisting for 12 months with an overall response rate of 90.9%, significantly higher than that observed in the control group (3). FMT alleviates pain through mechanisms involving modulation of gut microbiota and neuroinflammation. Specifically, FMT exerts analgesic effects by promoting the proliferation of anti-inflammatory bacterial species (e.g., Bifidobacterium and Lactobacillus) while reducing levels of pro-inflammatory cytokines, including IL-6 and TNF-α (109); FMT modulates levels of tryptophan metabolites (e.g., kynurenic acid and 3-indoxyl sulfate) and neurotransmitters (e.g., 5-HT and GABA), thereby regulating neuroinflammation and pain perception (110); FMT attenuates neuroinflammation and pain hypersensitivity by modulating gut microbiota composition and suppressing pro-inflammatory microglial activation in the CNS (12). In two clinical trials, the investigators intervened with FMT in 55 patients with fibromyalgia as well as 40 patients with IBS pain, and the results of the studies demonstrated that FMT significantly reduced pain scores (↓40%) and elevated pain thresholds (↑45%) in patients with fibromyalgia and IBS, accompanied by neurotransmitter improvements (↑28% for 5-HT, ↑31% for GABA) and inflammation suppression (TNF-α ↓36%), with efficacy lasting ≥12 months (3, 12).

### 6.3 Dietary modification (high fiber diet) enhances the antinociceptive effect of SCFA

High-fiber dietary regimens modulate gut microbiota composition to enhance the production of short-chain fatty acids (SCFAs, e.g., acetic acid, propionic acid, and butyric acid), which are critical for maintaining intestinal barrier integrity. This mechanistic cascade effectively attenuates pro-inflammatory mediators and nociceptive signaling, with long-term implementation demonstrating significant clinical improvements in osteoarthritic knee pain, functional disability, and pain duration (111). High-fiber dietary interventions promote the proliferation of beneficial bacterial species (e.g., *Bifidobacterium* and *Lactobacillus* spp.) while suppressing potential pathogens. The resultant SCFAs attenuate inflammatory responses through inhibition of NF-κB signaling pathway activation, thereby reducing the release of pro-inflammatory cytokines including IL-6 and TNF-α (112, 113), reduce systemic inflammatory response. SCFAs exert analgesic effects by modulating neurotransmitter levels, including 5-HT and GABA, thereby suppressing nociceptive signaling pathways (114). SCFAs attenuate nociceptive signaling through vagus nerve activation and functional modulation of the CN), thereby suppressing pain perception (115). High-fiber diets attenuate inflammation and pain by modulating gut microbiota composition and enhancing SCFA production. In a clinical trial of 214 patients with knee osteoarthritis and the NHANES cohort study of 1,892 personnel, the researchers used a high-fiber diet as an entry point for a related study, which found that a high-fibre diet (≥30 g/day) significantly improved osteoarthritis pain (WOMAC scores ↓35%), elevated levels of SCFAs (butyric acid ↑42%), and reduced systemic inflammation (IL-6↓29%, CRP↓26%), and long-term intake reduced chronic pain risk by 33% (116, 117). These microbial metabolites exert anti-nociceptive effects through dual mechanisms: inhibiting pro-inflammatory cytokine release (e.g., IL-6, TNF-α) and regulating neurotransmitter homeostasis (5-HT/GABA), thereby offering novel therapeutic strategies for pain management (Table 4).

**Table 4:** Microbiota-Targeted interventions for pain management.

Intervention	Mechanism	Clinical outcomes	References
Probiotics	Restore intestinal flora balance (e.g., increase Bifidobacteria, Lactobacillus) Inhibit the release of pro-inflammatory factors (IL-6, TNF-α) Regulate enteric nervous system function and inhibit injurious signaling	30%–50% reduction in postoperative pain scores (abdominal/orthopedic surgery patients) Decrease in pro-inflammatory factor levels 38%–41% (IL-6, TNF-α) Opioid dependence reduced by 35%	(50, 106–108)
Fecal microbiota transplantation (FMT)	Increases anti-inflammatory flora (e.g., Bifidobacteria) Increases serum 5-HT and GABA levels Reduces glutamate levels Inhibits microglia activation	40% reduction in pain scores (fibromyalgia/IBS patients) Pain threshold improved by 45 Neurotransmitter improvement (5-HT ↑28%, GABA ↑31%) Inflammation suppression (TNF-α ↓36%) Duration of efficacy ≥12 months (overall efficacy rate 90.9%)	(3, 12, 109, 110)
High-fiber diet	Promotes the production of SCFAs (butyric acid, propionic acid, acetic acid) Inhibits NF-κB signaling pathway and reduces pro-inflammatory factors (IL-6, TNF-α) Regulates neurotransmitters (5-HT, GABA)	Improved osteoarthritis pain (WOMAC score ↓ 35%) Elevated levels of SCFAs (butyric acid ↑42%) Reduced systemic inflammation (IL-6 ↓29%, CRP ↓26%) Reduced risk of chronic pain 33% with long-term intake	(111–117)
Butyrate supplementation	Inhibits histone deacetylase (HDAC) and reduces TNF-α production Stabilizes enteric nervous system activity Modulates central pain signaling via vagus nerve activity.	Preclinical models show neuropathic pain reduction (quantitative data pending clinical translation) Potential for postoperative pain management (mechanism support)	(6, 95)

Moreover Subgroup analyses suggest that probiotic efficacy in pain reduction may be more pronounced in females, possibly linked to estrogen-mediated modulation of gut barrier integrity and immune cell function (118). Future trials should stratify outcomes by sex to optimize personalized interventions.

## 7 Limitations and future directions

While this review synthesizes critical advances in gut microbiota-mediated pain sensitization, several limitations necessitate further investigation. Building on the future priorities outlined in the abstract, we propose the following expanded research agenda.

### 7.1 Causal validation beyond correlation

Current Limitation: Most evidence derives from correlative studies (e.g., dysbiosis-pain associations), lacking direct causal links between specific microbial taxa and pain phenotypes.

Future Pathways: Utilize germ-free animal models colonized with defined bacterial consortia (e.g., Clostridium scindens for bile acid metabolism) to establish microbial causality. Employ bacterial genetic engineering (e.g., CRISPR-Cas knockout of SCFA-producing genes) to dissect metabolite-specific effects on GPR43/TLR2 pathways.

### 7.2 Clinical translation of preclinical findings

Current Limitation: Heterogeneity in patient genetics, diet, and comorbidities limits generalizability of microbiota-targeted therapies (e.g., FMT/probiotics).

Future Pathways: Conduct large-scale, stratified RCTs focusing on pain subtypes (e.g., neuropathic vs. inflammatory pain) with multi-omics profiling (metagenomics, metabolomics) to identify predictive biomarkers. Develop standardized intervention protocols: Define optimal probiotic strains (e.g., *Bifidobacterium longum* for GABA modulation), FMT donor criteria, and high-fiber diet compositions.

### 7.3 Spatiotemporal dynamics of microbial signaling

Current Limitation: The temporal resolution of metabolite-receptor interactions (e.g., SCFA-GPR43 vs. LPS-TLR4 crosstalk) and their spatial distribution in neural tissues remain uncharacterized.

Future Pathways: Apply single-cell spatial transcriptomics to map microbial metabolite receptors (e.g., GPR43, TLR2) in DRG neurons and spinal glia. Utilize real-time metabolomic imaging (e.g., DESI-MSI) to track microbiota-derived molecules (e.g., KYNA, indole) across the gut-brain axis during pain progression.

### 7.4 Personalized microbial therapeutics

Current Limitation: Host-specific factors (e.g., epigenetics, immune status) influence treatment response, yet precision frameworks are underdeveloped.

Future Pathways: Integrate multi-omics data (host genomics, gut metagenomics, serum metabolomics) to build machine-learning models predicting individual responses to probiotics/FMT. Explore synbiotic formulations: Combine strain-specific probiotics (e.g., *Lactobacillus rhamnosus* GG) with prebiotics targeting metabolite production (e.g., resistant starch for butyrate). Integrate sex as a biological variable in multi-omics models to decipher hormone-microbiome-neuroimmune interactions underlying pain disparities.

### 7.5 Long-term safety and mechanistic depth

Current Limitation: Longitudinal effects of microbiota modulation (e.g., FMT-induced neuroimmune changes) are poorly documented.

Future Pathways: Establish long-term registries for patients receiving microbiota-based therapies, monitoring neurological/immunological outcomes (e.g., microglial activation via PET imaging). Decipher receptor crosstalk mechanisms (e.g., GPR43-TLR4 synergy) using organoid models or microfluidic gut-brain axis platforms.

## 8 Summary and perspectives

This review systematically consolidates the mechanisms underlying microbiota metabolite-mediated peripheral and central sensitization, establishing an integrative framework for understanding microbiome-pain interactions. Gut microbiota-derived metabolites, such as SCFAs, bile acids, and kynurenic acid, modulate sensitization processes in both central and peripheral nervous systems through diverse signaling pathways, including GPR43, TLR2, ASICs. Furthermore, gut dysbiosis exacerbates pain by promoting neuroinflammation and dysregulated immune responses. Microbiome-targeted interventions—including probiotics, fecal microbiota transplantation, and high-fiber diets—demonstrate therapeutic potential for pain management, offering novel strategies for future treatment.
